# Parent–adolescent communication in a digital world: A 100‐day diary study

**DOI:** 10.1111/cdev.14203

**Published:** 2024-11-22

**Authors:** Loes H. C. Janssen, Ine Beyens, Nadia A. J. D. Bij de Vaate, Amber van der Wal, Patti M. Valkenburg, Loes Keijsers

**Affiliations:** ^1^ Amsterdam School of Communication Research (ASCoR) University of Amsterdam Amsterdam The Netherlands; ^2^ Department of Psychology, Education, and Child Studies Erasmus University Rotterdam Rotterdam The Netherlands

## Abstract

Digital technology enables parents and adolescents to communicate anywhere and anytime. Knowledge of parent–adolescent online communication, however, is mainly based on cross‐sectional studies. In this preregistered 100‐day diary study, 479 adolescents (*M*_age_ = 15.98, 54.9% girls; 96.9% Dutch) reported daily if they had communicated with their parents online, how long (i.e., duration), and what they discussed (i.e., topics). Parent–adolescent online communication took place on 43% of days, for an average of 20 min a day, and predominantly concerned micro‐coordination. Five profiles of parent–adolescent online communication were identified, with most adolescents (55.4%) communicating relatively infrequently and briefly. Boys and younger adolescents communicated longer than girls and older adolescents. Although parent–adolescent online contact is possible all day, very few adolescents do so.

**Abbreviations**
BIC: Bayesian information criterion
LL: Loglikelihood
LMR‐LRT: Lo–Mendell–Rubin adjusted Likelihood Ratio Test
LPA: Latent profile analysis
RMLPA: Repeated measures latent profile analysis

Digital technologies such as smartphones have become integral to families' lives, facilitating adolescents and parents to communicate anywhere and anytime (e.g., Pew Research Center, 2018). The smartphone not only enables parents to check in on and control what their adolescents are doing and where they are but also offers them the potential of continuous supportive communication, also known as connected presence (Licoppe, 2004; Ribak, 2009). Hence, the smartphone could be considered a digital leash (i.e., a tool for control) as well as a digital umbilical cord (i.e., a lifeline that nourishes and protects), potentially having both negative and positive consequences.

On the one hand, the smartphone may inhibit adolescents' autonomy and be a digital pathway for overinvolvement and overparenting as parents are able to exert control over adolescents, even when they are not physically present (Chang, 2015; LeMoyne & Buchanan, 2011; Racz et al., 2015; Ribak, 2009). On the other hand, it may facilitate family connection and provide opportunities for nourishment as adolescents can ask for and parents can provide support digitally (e.g., LeMoyne & Buchanan, 2011; Ling, 2004; Walker & Rudi, 2014; Weisskirch, 2009, 2011). Even though these dynamics have become an important challenge in the relationship between parents and adolescents, there is a remarkable lack of scientific insights to provide evidence‐based advice on what it may mean to be able to communicate anywhere and anytime.

To better understand the potential consequences of parent–adolescent online communication, it is important to first assess how often and how long adolescents and parents engage in such communication. Most of the small body of work on parent–adolescent online communication is cross‐sectional, focusing on how often (i.e., frequency) parent–adolescent online communication occurs. These studies showed that 48% to 62% of adolescents reported texting their parents at least once a day (e.g., Chang, 2015; Lenhart et al., 2010; Padilla‐Walker et al., 2012). In some qualitative studies, adolescents were asked what they communicate about online with their parents (e.g., Fletcher et al., 2018; Racz et al., 2015; Tulane et al., 2022). Generally, these studies found that online parent–adolescent communication concerned managerial content (i.e., asking or answering questions) or emotional content (i.e., about feelings or experiences; Fletcher et al., 2018). A few observational studies examined the number of texts sent between adolescents or emerging adults and parents over a few days and coded their content, indicating that they texted with parents multiple times a day, mostly about positive or neutral content (Ehrenreich et al., 2020; Fletcher et al., 2018; Jensen et al., 2023; Jensen, Hussong, et al., 2021).

Altogether, research so far has mainly focused on assessing either the frequency or the topics of parent–adolescent online communication at one time point or qualitatively. However, an important piece of insight that is missing is how this communication unfolds in everyday life. Adolescents and parents have the possibility to connect, but it is unclear how often it happens throughout consecutive days and whether it differs between families. For instance, whether adolescents live with both parents, their age, or level of autonomy may impact both the frequency and duration of online communication between parents and adolescents. Therefore, the current 100‐day diary study aimed to obtain ecologically valid insights into the frequency, duration, and topics of parent–adolescent online communication, entailing chatting via WhatsApp, Snapchat, or Instagram, and understand how patterns of online communication with parents may differ across adolescents. This descriptive approach can serve as an important foundation to ultimately investigate the potential impact of these patterns on adolescents' well‐being.

## The frequency, duration, and topics of parent–adolescent online communication

A burst of research has underscored the importance of *offline* communication for the parent–adolescent relationship (Finkenauer et al., 2002; Kerr et al., 1999). Although exact insights into how often parents and adolescents communicate offline in daily life are scarce (Keijsers et al., 2022), studies suggest that they spend multiple hours a day together (Keijsers et al., 2010), creating ample opportunities for offline communication. Yet, as adolescents become more autonomous (e.g., Erikson, 1968; Soenens et al., 2019), the frequency of parent–adolescent offline communication is subject to change (e.g., Branje et al., 2012; Lionetti et al., 2019). For instance, how often parents and adolescents communicate decreases throughout adolescence (Keijsers & Poulin, 2013). However, whether adolescents' age and level of autonomy impact how they communicate *online* (both frequency and duration) with parents is still unknown.

The topics of parent–adolescent offline communication also change throughout adolescence: Parental knowledge of adolescents' whereabouts, for instance, decreases (Keijsers & Poulin, 2013; Lionetti et al., 2019). Parental knowledge can be the result of parental monitoring, which refers to parents' active efforts to stay informed about and keep track of what adolescents do, where they are, and with whom (Dishion & McMahon, 1998; Smetana, 2017; Stattin & Kerr, 2000). For example, parents may solicit information from their adolescents by asking them questions (Stattin & Kerr, 2000). However, as adolescents become more active agents, parents' knowledge most often comes from adolescents' disclosure of information (Stattin & Kerr, 2000). Adolescents may play an even more active role in *online* communication in that they can decide whether and when they respond to their parents' messages (Ling & Yttri, 2002; Ribak, 2009). Although the co‐construction theory indicates that offline and online communication can mirror or impact each other (Subrahmanyam et al., 2006), it is still an open question whether these findings on topics of offline communication are instrumental for understanding how parents and adolescents communicate online with each other, which is addressed in the current study on *online* communication.

Several quantitative studies have examined how often parents and adolescents communicate online, but most used a cross‐sectional design (e.g., Chang, 2015; Jensen, George, et al., 2021; Lenhart et al., 2010; Manago et al., 2020), which may result in biased insights due to recall. To overcome this bias, one observational study among emerging adults examined texting frequency with parents (Jensen, Hussong, et al., 2021). Results indicated that emerging adults, many of whom resided on college campus, exchanged approximately 10 text messages a day with their parents. Recently, two daily dairy studies (Jensen, George, et al., 2021; Manago et al., 2020) assessed parent–adolescent digital exchanges in daily life. One study indicated that adolescents communicated online with their parents on 29% of the days (Jensen, George, et al., 2021). The other study showed that parents and adolescents exchanged approximately nine texts a day and that they did so for approximately 15 min a day (Manago et al., 2020). Although these two daily diary studies examined the frequency and duration of parent–adolescent online communication (Jensen, George, et al., 2021; Manago et al., 2020), their measures included chatting and calling and made no distinction between these two communication modes. Adolescents indicated in earlier work that they preferred chatting over calling (e.g., Blair et al., 2013; Racz et al., 2015). Therefore, the current study solely focused on chatting.

With regard to topics of parent–adolescent online communication, mostly qualitative evidence showed that parental solicitation and adolescent disclosure occur, at least partly, online (e.g., Fletcher et al., 2018; Racz et al., 2015; Tulane et al., 2022). For instance, one qualitative study interviewed 14 adolescents (14–18 years old) and showed that most parent–adolescent online communication entailed managerial communication, including discussing mundane activities, such as logistics, monitoring location, planning activities, or asking and answering practical questions (Fletcher et al., 2018). Such parent–adolescent interaction is also known as micro‐coordination, referring to communication that entails the coordination of functional and instrumental needs, often with a social and caring function (Ling & Yttri, 2002). Parent–adolescent online communication also involved emotional connection, which refers to communicating about and elaborating on one's experiences or feelings. However, this type of online communication occurred less frequently (Fletcher et al., 2018). Another study coded messages exchanged between parents and adolescents but focused more on the valence of these messages (i.e., positive, neutral, and negative) rather than their topics (Ehrenreich et al., 2020).

Altogether, a handful of empirical quantitative research has provided a glimpse into how frequently and long the average adolescent communicates with their parents online. Moreover, qualitative interview studies provided a general idea of the topics adolescents and parents communicate about online from adolescents' perspective. However, both approaches may be prone to recall bias and a more nuanced understanding of how adolescents and parents navigate the introduction of smartphones and continuous connectivity in their daily lives is still hardly understood. In cross‐sectional designs, adolescents may over‐ or underestimate how often and how long parent‐adolescent online communication takes place. In interviews or focus groups, adolescents may forget to report topics that are not discussed that frequently or provide socially desirable answers. The use of a 100‐day diary study can overcome these limitations and ensure a more ecologically valid assessment of the frequency, duration, and topics of parent–adolescent online communication. Thus, we investigated the following research question:

(RQ1) How often (i.e., frequency), how long (i.e., duration), and about what (i.e., topics) do adolescents and parents communicate online on a daily level?

## Profiles of parent–adolescent online communication

Even though almost all adolescents nowadays carry a smartphone, which provides them with the opportunity to stay in contact with their parents, not all adolescents are connected with their parents to the same extent. In the last few decades, it has been increasingly acknowledged in developmental psychology that the average adolescent does not exist (e.g., Granic et al., 2003). Recent work has also highlighted substantial differences between adolescents in social media use and showed that a classification can help to better understand these differences (Beyens et al., 2021). A similar approach can be used to enhance our understanding of parent–adolescent online communication. For example, Charlie may have daily but short online communication with his parents, while Sam may have infrequent but longer online communication with her parents. This may lead to the same average levels of communication, albeit with two different underlying communication profiles. Identifying groups of adolescents with similar profiles of parent–adolescent online communication using both frequency and duration of communication can help to gain a first insight into which parent–adolescent online communication behaviors exist.

A person‐centered approach, such as latent profile analysis (LPA), enables classifying adolescents into meaningful groups based on response patterns of variables (Jung & Wickrama, 2008), such as frequency and duration of parent–adolescent online communication. Based on results of three previous studies on parent–adolescent online communication, with a focus on the frequency and coded content, three distinct profiles can be distinguished. In two studies, approximately 50% of adolescents communicated daily with their parents online, and 25% of adolescents never did so (e.g., Jensen, George, et al., 2021; Padilla‐Walker et al., 2012). Additionally, a study among emerging adults introduced further differentiation between infrequent and frequent communication (Jensen, Hussong, et al., 2021). Together, these findings suggest that (at least) three profiles of parent–adolescent online communication may exist (daily/frequent, infrequent, and no parent–adolescent online communication). However, in the absence of work that combines both frequency and duration, we could not hypothesize the exact number of profiles. Therefore, we explored the following research question:

(RQ2) How many and which profiles of parent‐adolescent online communication exist?

## Explaining differences between profiles of parent–adolescent online communication

Individual characteristics may explain why patterns of online communication with parents of adolescents like Sam and Charlie may differ. The differential susceptibility to media effects model (Valkenburg & Peter, 2013) distinguishes three types of factors that may influence the use of media: dispositional, developmental, and social‐context factors. However, thus far, it is not known whether these factors also influence *both* how long and frequent adolescents and parents communicate online with each other. The limited research on parent–adolescent online communication mainly focused on the frequency or content of this communication rather than duration (e.g., Fletcher et al., 2018; Jensen, George, et al., 2021). Yet, some first insights indicate that gender, autonomy, age, and family living situation are of particular interest and may be linked to patterns of communication.

With regard to gender, girls seem to have more frequent online communication with their parents than boys (Ehrenreich et al., 2020; Fletcher et al., 2018; Jensen, George, et al., 2021; Padilla‐Walker et al., 2012). However, the duration of this communication does not differ between boys and girls (Manago et al., 2020). Regarding autonomy, there is a lack of consensus on whether parent–adolescent online communication may inhibit (Racz et al., 2015) or stimulate adolescent autonomy (Manago et al., 2020). According to the hyperpersonal model, online communication can enhance feelings of agency (Walther, 1996) as adolescents can choose when and how to engage in these online interactions. Adolescents' feelings of autonomy may therefore impact how often and how long adolescents communicate online with their parents. Concerning age, parent–adolescent online communication tends to increase across adolescence until the age of 16/17 and thereafter decreases again (Jensen, George, et al., 2021; Padilla‐Walker et al., 2012). However, the duration of this communication does not differ based on age (Manago et al., 2020). With regard to living situation, when adolescents do not live with (both) parents, parent–adolescent online communication may be a convenient way to stay in contact with the parent who is physically absent. A previous study indeed found that single‐parent families used parent–adolescent online communication (both calling and texting) more than two‐parent families (Padilla‐Walker et al., 2012). Whether the duration is also impacted by the living situation remains the question. Based on these first insights, we explore the predictive role of two dispositional factors (i.e., gender and autonomy), one developmental factor (i.e., age), and one social‐context factor (i.e., family living situation) on how adolescents differ in both the frequency and duration of parent–adolescent online communication (i.e., profiles), by investigating the following research question:

(RQ3) Do the distinct parent‐adolescent online communication profiles differ by gender, autonomy, age, and family living situation?

## The current study

In order to better understand how adolescents and parents deal with the possibility of connecting online all day and every day, this study fills an important lacuna by providing descriptive insights into parent–adolescent online communication in daily life. This study is based on a daily diary study in which 480 adolescents (14–17 years old) were meticulously followed for 100 days and completed a questionnaire every day. Three research aims were preregistered to extend the available body of research (https://osf.io/9vyfr). First, we examined how often, how long, and about what topics adolescents and parents communicated online over a period of 100 days in an ecologically valid way. Second, we investigated whether certain profiles of parent–adolescent online communication exist, based on the frequency and duration of such communication, to gain a more nuanced understanding of adolescents who communicate with their parents online. Third, in order to understand differences between the profiles, we examined whether derived profiles of parent–adolescent online communication differ in age, gender, family living situation, and autonomy of adolescents. Due to the limited research available, this study is exploratory and no hypotheses were specified.

## METHOD

This study is part of a larger intensive longitudinal project that investigates the effects of social media use on various aspects of adolescent well‐being. Data collection took place from January 2023 to June 2023 and included four phases: an online intake interview (I), a baseline questionnaire (II), a 100‐day daily diary (III), and an optional exit interview to give adolescents who expressed interest insights into their social media patterns (VI; see https://osf.io/k47ta for a full overview of the project's procedure). This study used data from the baseline questionnaire and the 100‐day daily diary study.

### Participants

A total of 480 adolescents, aged 14 to 17 years at the time of inclusion, from all regions (both rural and urban) of the Netherlands participated in the larger project. Due to personal circumstances, one adolescent stopped participating in the daily diary after the first day and was therefore excluded from this study. Thus, the final sample consisted of 479 adolescents (*M*_age_ = 15.98, SD = 1.15; 44.3% boys, 54.9% girls, 0.8% nonbinary). Participants were enrolled in different educational tracks: 143 participants (29.9%) in (pre)vocational education, 140 participants (29.2%) in higher general secondary/higher professional education, and 196 participants (40.9%) in (preparatory) academic education. Most adolescents (96.9%) were born in the Netherlands and most (93.9%) self‐identified as Dutch. The majority of adolescents (74.3%) lived with both parents, a small group alternated between parents (9.6%), or lived only with their mother (7.3%), with their mother and partner (4.6%), only with their father (1.7%), with other educators (e.g., foster parents) (1.3%), with their father and partner (1.0%), or on their own (0.2%).

### Procedure

The larger project was approved by the Ethics Review Board of the Faculty of Social and Behavrioal Sciences at the University of Amsterdam (2022‐YME‐15724). Recommendations for collecting intensive longitudinal data were followed (van Roekel et al., 2019). Adolescents were involved in the design process of the study. Before the study started, five qualitative discussion sessions with a youth advisory panel of a total of nine adolescents (13–17 years old; 66.7% girls) were organized to discuss the study procedure, sampling scheme, and daily diary items. Next to this youth advisory panel, a 2‐week pilot test of our daily diary was held among 26 adolescents (13–17 years old; 50% girls).

The project aimed to include 400 adolescents in the 100‐day diary study. To take into account potential attrition, we planned to schedule around 500 adolescents for an intake interview. To realize a sample of adolescents representative of Dutch 14‐ to 17‐year‐olds in terms of gender, age, and educational track, we collaborated with research company CHOICE. Additionally, we recruited participants via advertisements in our personal network, social media, and from earlier projects. Information letters and videos about the aims and procedure of the study were provided on the project website. All adolescents, as well as parents of adolescents below the age of 16, provided informed consent. This resulted in 829 registrations, of which 480 adolescents participated in the larger study project (see https://osf.io/k47ta for more details on the youth advisory panel, pilot study, recruitment and intake procedure).

Before the intake interviews, adolescents were instructed to install the daily diary software application m‐Path (m‐path.io; Mestdagh et al., 2023) on their phones. During the intake interviews, we asked about adolescents' social media use, explained the procedure of the study, and provided instructions on how to use the m‐Path app. We also collaboratively completed a short survey in m‐Path to explain the different types of questions and response options. Adolescents were asked in this survey to select the three social media platforms they used most often. Five days before the 100‐day daily diary study started, participants received a link to the baseline questionnaire, including questions on background and personality characteristics. All 480 adolescents completed the baseline questionnaire.

#### Daily diary

During 100 consecutive days, from January to May 2023, participants received one questionnaire a day via the m‐Path app. The micro‐questionnaires were sent at 8.30 p.m. and could be started until midnight. Reminders were sent at 9.15 and 10.00 p.m. if adolescents had not completed the questionnaire. Each questionnaire consisted of 34 to 38 questions, depending on the number of follow‐up questions. After the 100 days, participants could decide to extend their participation up to 15 days to catch up for missed days.

We monitored participants' compliance daily and answered any participant questions or problems via WhatsApp, telephone, and e‐mail. Participants were messaged regularly via WhatsApp to motivate them. For instance, we updated them weekly on their response rates of the previous week. Generally, when participants missed three and four questionnaires in a row, we contacted them via WhatsApp to check whether they were experiencing any technical issues. If participants missed approximately a week of questionnaires, we called them to check whether there were any technical issues or whether we could help to motivate them.

#### Incentives

Adolescents received compensation for each phase of the study, except for the optional interview. Adolescents received €5,‐ for the intake interview, €5,‐ for completing the baseline questionnaire, and €1,‐ for each completed daily diary questionnaire. Participants who completed 100 questionnaires or more (including catch‐up days) received an additional €10,‐. In the middle of the study, participants could earn a €5,‐ bonus by completing 14 consecutive questionnaires in a row (Day 47 until 60). In addition, each Tuesday, we raffled two times €25,‐ based on compliance of the previous week. Participants were paid every month of the study.

#### Compliance

Across the 100 days, 82.8% of the sent daily diary questionnaires were completed (39,598 of 47,847 observations). After the 15 catch‐up days, adolescents had answered on average 92.3 daily questionnaires (SD = 24.55, range 12–115), resulting in 44,211 completed questionnaires. A small proportion of the daily diaries (115 questionnaires, <0.3%) had irregularities or were not sent due to unforeseen technical issues with the m‐Path application. Non‐compliance was partly due to human factors (e.g., not being able to use the m‐Path app because of parental punishment, being ill, or a phone dropped in the toilet) as well as technological factors (e.g., having no internet access or uploading errors).

### Measures

#### Frequency of parent–adolescent online communication

We assessed the frequency of parent–adolescent communication in the daily diary by asking participants “Did you chat with your parent(s) or guardian(s) today (via Snapchat, WhatsApp, or Instagram)?” Adolescents could respond with yes (1) or no (0). A person‐specific mean variable was calculated to represent how often adolescents communicated with their parents online throughout the study period.

#### Duration of parent–adolescent online communication

If adolescents indicated that they had chatted with their parents, a follow‐up question assessed the duration of chatting daily by asking: “How long did you chat with your parent(s) or guardian(s) today (via Snapchat, WhatsApp, or Instagram)?” Adolescents were instructed to provide an estimation in hours and minutes, which we recoded to minutes. Adolescents did not indicate having problems with answering this question as described in Appendix S1. A person‐specific mean variable was calculated to indicate how long, on average, adolescents communicated with their parents online throughout the study period.

#### Topics of parent–adolescent online communication

We assessed the topics adolescents and parents chatted about daily by asking participants a multiple‐response question: “What did you chat about with your parent(s) or guardian(s) today (via Snapchat, WhatsApp, or Instagram)?” We included seven a‐priori response categories: (1) groceries or food, (2) school, (3) sports, (4) who you are with, (5) where you are (i.e., whereabouts), (6) what time you get somewhere, (7) how you are doing, and (8) other. Adolescents could insert text when they selected “other”. The a‐priori response categories were based on open answers provided in our 14‐day pilot study among 26 adolescents (see Appendix S1 for more information) and by the parenting literature (Stattin & Kerr, 2000). These categories are also in line with those found in previous qualitative research (e.g., Fletcher et al., 2018). The text in the “other” category was inspected and recoded if it corresponded to an existing response category. For example, the answer “grades” in the “other” category was recoded to a‐priori category “school.” Based on the remaining open answers, additional categories were determined by the first author and discussed within the research team. The team agreed on the following seven additional categories: (8) monitoring activities (e.g., what are you doing), (9) practical issues (e.g., money, chores, doctors appointment), (10) entertainment (e.g., holiday, photograph's, music), (11) family issues (e.g., fight with father, work father), (12) adolescents monitoring their parents (e.g., asking when parents are at home), (13) adolescents supporting their parents (e.g., asking parents how they are doing), and (14) unspecified (e.g., private, nonsense, things that did not match previous categories and were not mentioned often). A count variable was calculated per category to represent how often each adolescent communicated with their parents online about that specific category across the 100 days.

#### Autonomy

To assess adolescents' sense of autonomy, the volition subscale of the Self‐Determination Scale (Sheldon et al., 1996) was administered in the baseline questionnaire. Volition refers to the extent to which someone feels a sense of freedom to make their own choices and decisions. The five items of the subscale (e.g., “I feel free to do the things I want to do”) were answered on a 5‐point scale ranging from 1 (*not at all true*) to 5 (*completely true*). Previous work has shown adequate psychometric properties in adolescent samples (e.g., Sheldon et al., 1996; Soenens et al., 2007). An exploratory factor analysis in R yielded one factor, which explained 40.5% of the variance (with all factor loadings ≥0.45). Cronbach's alpha was .77. A mean score was calculated with higher scores indicating higher levels of autonomy.

#### Descriptive variables

Measurement of age, gender, and family living situation assessed in the baseline questionnaire is presented in Appendix S2.

### Preregistered statistical analysis plan

We preregistered research questions and our analysis plan before analyzing the data (https://osf.io/9vyfr). To answer our first research question and investigate how often (i.e., frequency), how long (i.e., duration), and about what topics adolescents and parents communicated across the study period, we calculated descriptive statistics using R (version 4.2.2). Additionally, for descriptive purposes, we calculated the between‐person correlations between the person‐mean averages of frequency and duration of parent–adolescent online communication, age, gender, family living situation, and autonomy.

To answer our second research question and identify latent profiles in parent–adolescent online communication, we aimed to employ a repeated measures latent profile analysis (RMLPA) in M*plus* (Version 8.8) using both frequency and duration of parent–adolescent online communication. RMLPA has recently been applied to longitudinal data with three or 27 times points in their analyses (see May et al., 2020; McCarthy et al., 2016). Due to the richness of our data (115 observations of a continuous and dichotomous variable), computational power was lacking and our RMLPA models did not converge. Therefore, we decided to examine our research questions with a more parsimonious analytical approach. We used person‐mean variables of frequency and duration of parent‐adolescent online communication and applied established LPA (Jung & Wickrama, 2008).

We based the number of tested profile solutions partly on earlier empirical work which was mostly based on the frequency (and coded content) of parent–adolescent communication (Jensen, George, et al., 2021; Jensen, Hussong, et al., 2021; Padilla‐Walker et al., 2012). Studies suggested three distinct parent‐adolescent online communication profiles: no parent–adolescent online communication, infrequent parent–adolescent online communication, and frequent or daily parent‐adolescent online communication. In our data, *n* = 15 participants reported no parent–adolescent communication. In the absence of this communication with parents, the duration could not be assessed, which is why we could not include these participants in the profile analyses based on frequency and duration. As sensitivity analyses, we reran the LPA analyses with the 15 adolescents included, see Appendix S3 for the results.

For both the infrequent and frequent parent–adolescent online communication group, we suspected that the duration of parent–adolescent online communication may be high or low resulting in the emergence of four profiles in the profile analysis when including both frequency and duration of parent‐adolescent online communication; infrequent low duration communication, infrequent high duration communication, frequent low duration communication, and frequent high duration communication. Models were estimated in an iterative manner, starting with a one‐profile solution, and adding an extra profile in every step. We also tested a five‐ and six‐profile solution for transparency and controlling purposes (following Van De Schoot et al., 2017). The final solution of profiles was based primarily on BIC (Bayesian information criterion; lower values indicate better model fit) and secondarily in terms of the LMR‐LRT (Lo–Mendell–Rubin adjusted Likelihood Ratio Test) (following Van De Schoot et al., 2017). To interpret the quality of the chosen solution, the entropy index of model‐based classification accuracy was considered. Generally, values larger than 0.8 indicate adequate classification and values lower than 0.6 indicate misclassification (Lubke & Muthén, 2007). Other criteria were theoretical, including the research question, parsimony, theoretical justification, and interpretability (following Jung & Wickrama, 2008).

In the next step, we answered our third research question, by assessing whether four individual characteristics predicted profile membership regarding parent‐adolescent online communication. Here, we deviated from our preregistered approach to improve the quality of comparisons. Instead of saving profile membership and using ANOVA's and chi‐squared difference tests for testing differences between profiles, we used the 3‐step procedure in M*plus*, which allows accounting for uncertainty in profile membership (Asparouhov & Muthén, 2014; Vermunt, 2017). We ran four models, one for each predictor. Results of the preregistered analyses can be found in Appendix S4.

## RESULTS

### Descriptive statistics and correlations

Descriptive statistics and between‐person correlations are provided in Table 1. On average, adolescents who communicated more frequently with their parents online than their peers did so for a shorter duration. Adolescent girls had more frequent parent–adolescent online communication than boys, while boys tended to have longer communication. Adolescents who did not live with both of their parents communicated more often and longer online with their parents. Age and autonomy were not related to the frequency and duration of parent‐adolescent online communication.

**TABLE 1:** Descriptive statistics and between‐person correlations of study variables.

Variables	Descriptives				Between‐person correlations	
	*n*	*M* (SD)/Mdn	Min	Max	1	2
1. Frequency of communication percentage/days^a^	475	0.43 (0.32)/0.37	0.00	1.00		
2. Duration of communication minutes/day	460	19.55 (29.42)/9.59	0.00	237.00	−0.158***	
3. Age	475	15.97 (1.15)	14.01	18.11	−0.029	−0.072
4. Gender^b^	475	1.55 (0.50)	1	2	0.138**	−0.152**
5. Family living situation^c^	475	1.25 (0.43)	1	2	0.108*	0.094*
6. Autonomy	475	3.79 (0.58)	2.00	5.00	0.002	0.034

*Note*: Four adolescents (nonbinary) are not included to facilitate interpretation.

^a^ Person‐mean reflecting average percentage of days adolescents and parents communicated online.

^b^ Only boys (1) and girls (2) were included.

^c^ Family living situation was recoded to living with both parents (1) or other living situations (2).

* *p* < .05.

** *p* < .01.

*** *p* < .001.

### Frequency, duration, and topics of parent‐adolescent online communication

**FIGURE 1:**
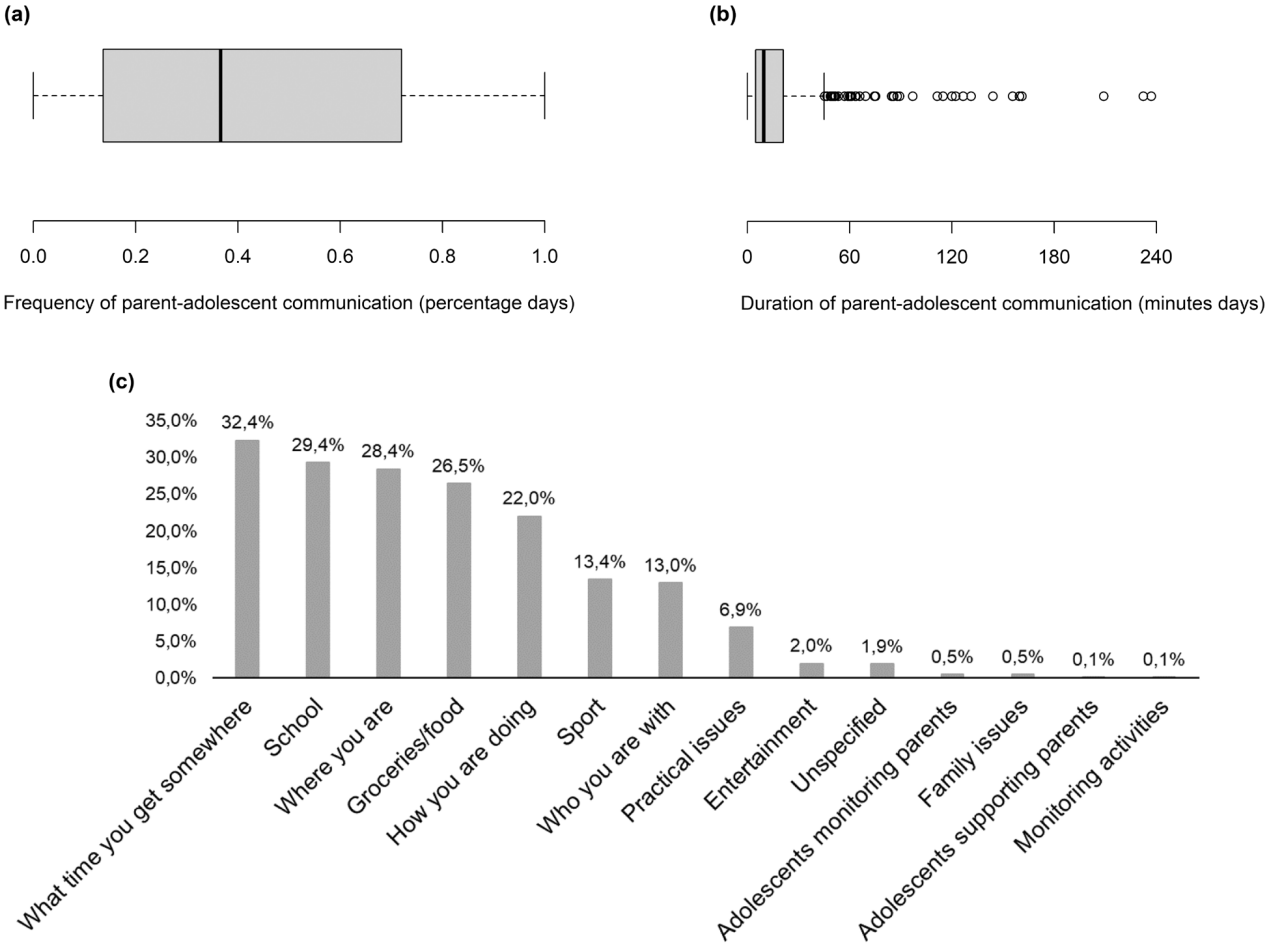
Distribution of frequency (a), duration (b), and topics (c) of parent–adolescent online communication.

Our first research question (RQ1) asked how often, how long, and about what topics adolescents and parents communicate online on a daily level. On average, adolescents communicated online with their parents on 43% of the days. See Figure 1a for the slightly skewed distribution of the person‐mean of frequency of parent–adolescent online communication. Except for 15 adolescents (3.1%), almost all adolescents (96.9%) communicated online with their parents. Of the 464 adolescents who communicated with parents online, eight adolescents (1.7%) did so every day and 67 (14.4%) communicated daily or almost every day (i.e., more than 90% of days). Adolescents who communicated with their parents online did so on average for almost 20 min per day, with half of adolescents communicating for more than 10 min and the other half for less than 10 min. This duration ranged from 0 min to almost 4 h on a given day. See Figure 1b for the skewed distribution of the person‐mean of duration of parent–adolescent online communication. There was more and longer parent‐adolescent communication on weekdays compared to weekend days (frequency: *χ* ^2^ (1) = 90.72, *p* < .001; *M*_week_ = 0.44, SD_week_ = 0.50, *M*_weekend_ = 0.39, SD_weekend_ = 0.49, duration: *H*(1) = 6.39, *p* = .011; *M*_week_ = 15.29, SD_week_ = 51.68, *M*_weekend_ = 14.68, SD_weekend_ = 47.37).

Figure 1c provides an overview of all topic categories covered in adolescents' online communication with their parents. The top 5 most discussed topics were: the time they got somewhere (32.4% of observed parent–adolescent online communication), school (29.4%), where they were (i.e., whereabouts 28.4%), groceries or food (26.5%), and how you are doing (22.0%). Most frequent topics concerned monitoring or micro‐coordination. The “how you are doing” category may entail support or emotional communication. New topics also emerged. For instance, some adolescents used parent‐adolescent online communication to monitor (0.5%) or support (0.1%) their parents. Adolescents could select multiple topics per day. On average, they discussed 1.77 topics (SD = 1.15, range 0–8) per day with parents online. In 336 observations, answers were missing due to a programming error that accidentally enabled adolescents to skip the question about the topics of their communication.

### Profiles of parent–adolescent online communication

Our second research question (RQ2) asked how many distinct profiles of parent‐adolescent online communication existed based on the frequency and duration of such communication. Participants were only included in the profile analysis if any parent‐adolescent online communication took place across the study period. In the profile analyses, default starting values were used, but the best loglikelihood value was not replicated in all models. Starting values were therefore increased. The best loglikelihood value was replicated in all profile solution models when using random start values 800 and 160.

We found five different profiles. Table 2 shows that the BIC of the five‐profiles solution was lower than the BIC of the four‐profiles solution and the LMR‐LRT confirmed that the five‐profiles solution was significantly better than the four‐profiles solution. Even though the six‐profiles solution had an even lower BIC, the LMR‐LRT was not significant, which indicates that this solution did not fit the data better than the five‐profiles solution. The quality of the five‐factor solution was assessed and entropy was good (above 0.80; Lubke & Muthén, 2007). A scree plot and figures of all other profile solutions can be found in Appendix S5.

**TABLE 2:** Fit measures latent profile analyses (*N* = 464 adolescents).

# of classes	BIC	AIC	LL (parameters)	LMR‐LRT (*p*)	Entropy	Number (percentage) per profile					
						1	2	3	4	5	6
1	4730.829	4714.270	−2353.135	‐	‐	464 (100%)					
2	4399.728	4370.749	−2178.375	331.522 (.524)	0.990	447 (96.3)	17 (3.7%)				
3	4275.693	4234.295	−2107.147	135.119 (.214)	0.970	410 (88.4%)	41 (8.8%)	13 (2.8%)			
4	4137.896	4084.078	−2029.039	148.173 (.011)	0.985	406 (87.5%)	44 (9.5%)	11 (2.4%)	3 (0.6%)		
5	**4046.707**	**3980.469**	**−1974.234**	**103.965 (.002)**	**0.923**	**257 (55.4%)**	**155 (33.4%)**	**39 (8.4%)**	**10 (2.2%)**	**3 (0.6%)**	
6	4016.541	3937.883	−1949.941	46.084 (.118)	0.922	253 (54.5%)	148 (31.9%)	42 (9.1%)	10 (2.2%)	8 (1.7%)	3 (0.6%)

*Note*: Class counts and proportions are based on their most likely class membership. Stable class solution was tested by 800 and 160 random starts. To test the stability of the chosen solution, models were run again with doubled starting values (1600 and 320) and model results were replicated. Bolded values represent the final profile solution (based on LMR‐LRT).

Abbreviations: AIC, Akaike information criterion; BIC, Bayesian information criterion; LL, Loglikelihood; LMR‐LRT, Lo–Mendell–Rubin adjusted Likelihood Ratio Test.

**FIGURE 2:**
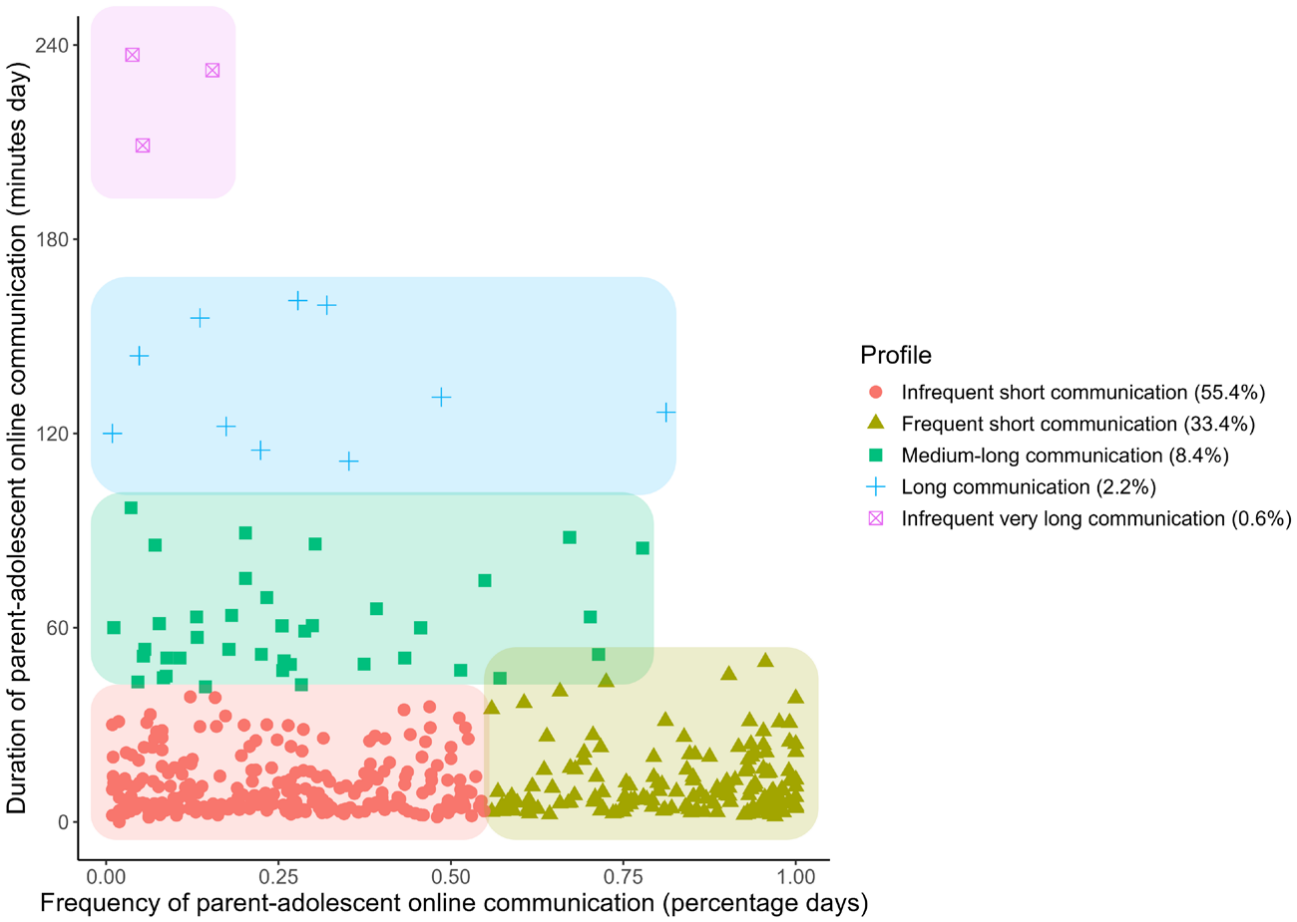
Five profiles of parent–adolescent online communication.

The five distinct profiles are presented in Figure 2. We labeled the profiles relative to each other (i.e., short, long and infrequent, frequent relative to the other profiles). The biggest group, 55.4% of adolescents (*n* = 257), communicated online with their parents for less than half of the days and less than an hour a day. This profile was therefore labeled *infrequent short communication*. Another large group composed of 33.4% of adolescents (*n* = 155) communicated online with their parents on more than half of the days and did so less than an hour a day. Hence, this group of adolescents was labeled *frequent short communication*. A smaller group, 8.4% of adolescents (*n* = 39), did not communicate online with their parents on all days but if they communicated, they did between approximately 1 and 2 hours. This profile was labeled *medium‐long communication*. A small group, 2.2% of adolescents (*n* = 10; which we labeled *long communication*), did not communicate online with their parents on all days but if they communicated, they did between 2 or 3 hours a day. Finally, a very small group of 0.6% of adolescents (*n* = 3) had *infrequent very long communication*. They communicated online with parents for less than a quarter of the days and between 3 and 4 hours a day.

### Differences in patterns of parent–adolescent online communication

Our third research question asked whether gender, autonomy, age, and family living situation could predict profile membership (RQ3). Comparisons between all five profiles for all four predictors are presented in Table 3. We had preregistered to interpret differences between profiles only when profiles included 40 or more adolescents (based on our power analysis). As one profile had 39 adolescents, which is a minor deviation from that cut‐off, we decided to take this profile into account. The two smallest profiles were not considered for interpreting differences due to their small size. Hence, we only interpreted differences between adolescents in the three largest profiles: adolescents with *infrequent short communication*, adolescents with *frequent short communication*, and adolescents with *medium‐long communication*. The model including gender only included boys and girls, as the non‐binary category was too small to be included (4 persons).

**TABLE 3:** Profile differences predicting by age, gender, autonomy, and living situation.

	Age	Gender^a^	Autonomy	Living situation
	Est (*p*‐value)	Est (*p*‐value)	Est (*p*‐value)	Est (*p*‐value)
Infrequent short versus frequent short	−0.101 (.304)	0.147 (.529)	0.130 (.505)	0.354 (.170)
Infrequent short versus medium‐long	**−0.401 (.015)**	**−0.804 (.030)**	0.458 (.166)	0.423 (.284)
Infrequent short versus long	−0.276 (.416)	−0.721 (.274)	−0.581 (.395)	0.826 (.214)
Infrequent short versus infrequent very long	−0.234 (.673)	**−19.058 (<.001)**	1.475 (.123)	0.538 (.663)
Frequent short versus medium‐long	−0.300 (.776)	**−0.951 (.014)**	0.328 (.338)	0.069 (.865)
Frequent short versus long	−0.175 (.609)	−0.868 (.195)	−0.711 (.303)	0.473 (.482)
Frequent short versus infrequent very long	−0.133 (.811)	**−19.204 (<.001)**	1.345 (.162)	0.185 (.881)
Medium‐long versus long	0.125 (.736)	0.083 (.911)	−1.039 (.172)	0.403 (.588)
Medium‐long versus infrequent very long	0.166 (.771)	**−18.254 (<.001)**	1.017 (.306)	0.116 (.928)
Long versus infrequent very long	0.042 (.949)	**−18.336 (<.001)**	2.056 (.080)	0.538 (.663)

*Note*: The first category that is mentioned is the reference category. Bolded values indicate significant differences.

^a^ Model only included boys and girls to resemble original analysis.

**FIGURE 3:**
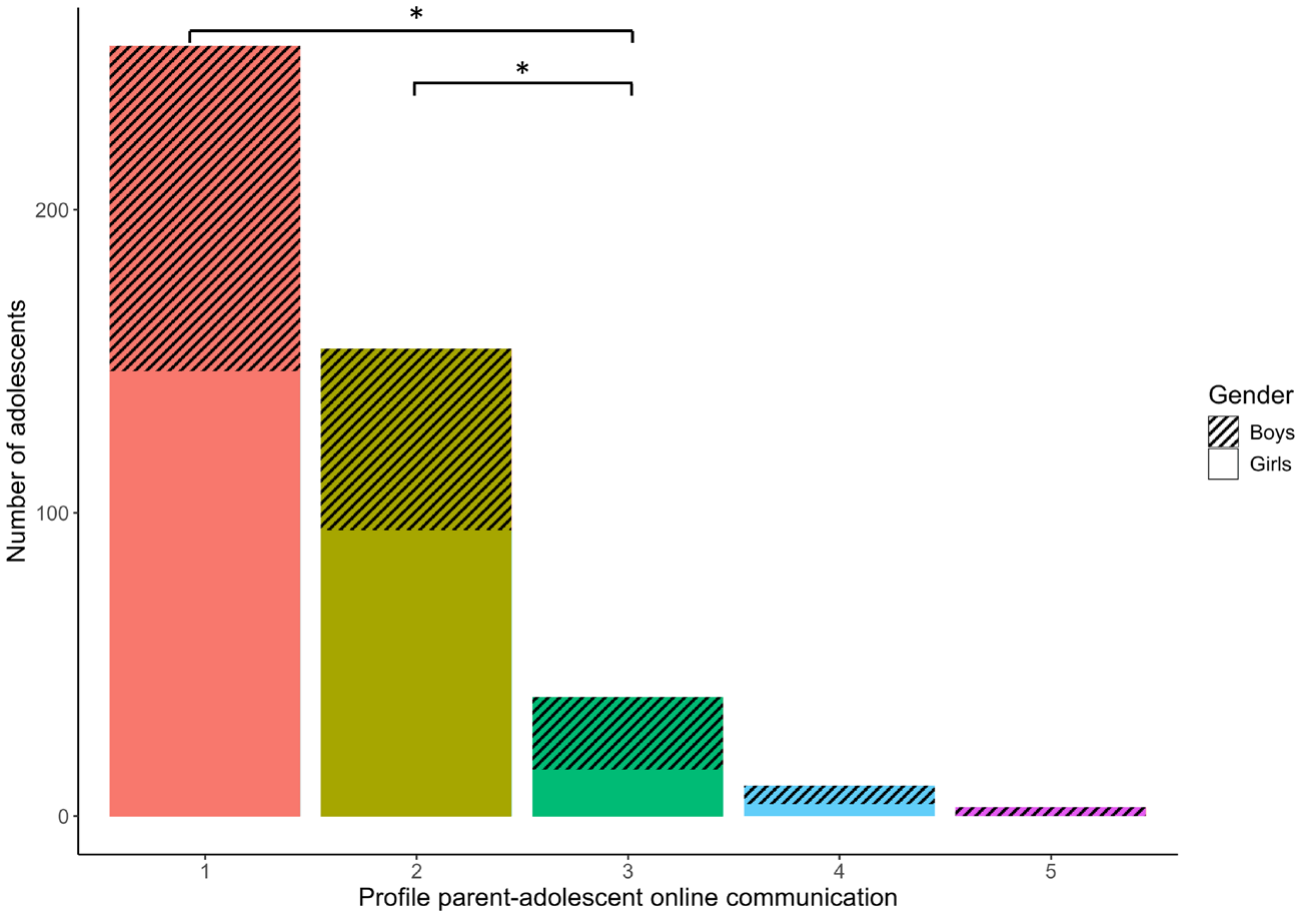
Gender predicting profile membership, with more adolescent boys in profile 3 (medium‐long communication) than in profile 2 (frequent short communication) and profile 1 (infrequent short communication). * *p* < .05.

**FIGURE 4:**
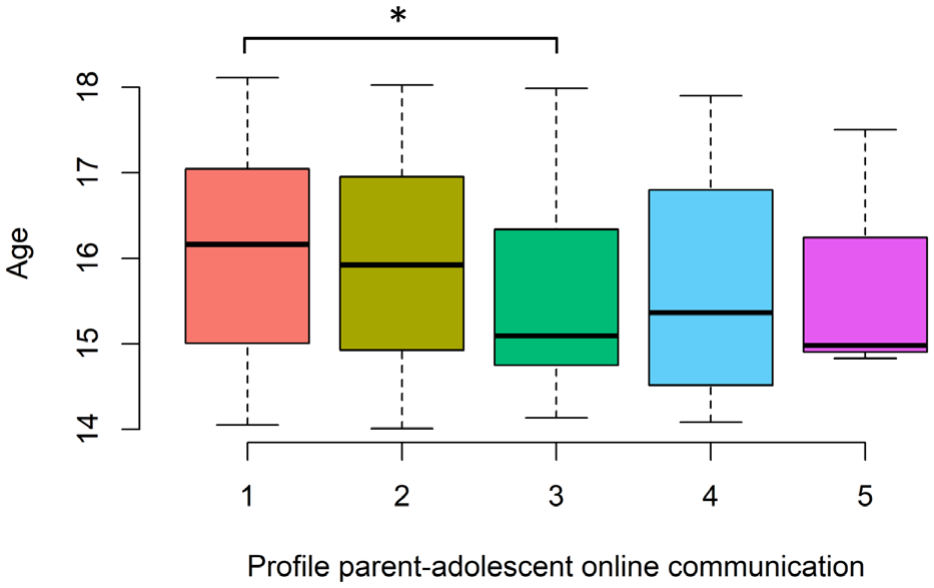
Age predicting profile membership, adolescents in profile 3 (medium‐long communication) were younger than adolescents in profile 1 (infrequent short communication). * *p* < .05.

Only age and gender were significant predictors of profile membership. Adolescents with *medium‐long communication* were younger than adolescents with *infrequent short communication* (Figure 3), indicating that younger adolescents communicated longer with parents online than older adolescents. Adolescents with *medium‐long communication* were also more likely to be boys than adolescents with *infrequent short communication* and with *frequent short communication* (Figure 4), suggesting that boys tend to engage in longer online communication with parents than girls.

### Exploratory analysis

In order to explore whether topics of parent–adolescent online communication differed across profiles, we calculated how often adolescents communicated online with their parents about the topics for the three largest profiles separately. The top three most discussed topics differed slightly across the profiles. Adolescents with *infrequent short communication* communicated most frequently about what time they got somewhere, where they were (i.e., whereabouts), and school. Adolescents with *frequent short communication* communicated most frequently about what time they got somewhere, school, and food. Adolescents with *medium‐long communication* communicated most frequently about school, where they were (i.e., whereabouts), and what time they got somewhere.

### Sensitivity analyses

To assess the robustness of our findings, we ran two sets of sensitivity analyses. First, we checked the potential influence of flagged observations on the profile solution. Second, we treated autonomy as distal outcome and included gender, age, and family living situation as covariates to account for demographic differences (Vermunt, 2017). A detailed overview of the sensitivity analyses and results can be found in Appendix S6. All results and conclusions remained virtually unchanged. Age and gender did no longer predict profile membership of parent‐adolescent online communication when excluding flagged observations.

## DISCUSSION

Digital technologies have transformed parent–adolescent communication, enabling it to take place anytime and anywhere (e.g., Pew Research Center, 2018). The question remains whether families also engage in such communication on a daily basis and, if so, if it is impacted by factors like age and living situation. The current study is one of the first to investigate the frequency, duration, and topics of parent‐adolescent online communication over an extended period of time, using a 100‐day diary study with 479 adolescents. The results, based on 18,930 observations, demonstrated that, overall, adolescents communicated online with their parents on 43% of the days, with half of adolescents communicating more than 10 min and the other half less than 10 min, but this duration varied across days and among adolescents. Their online communication mostly concerned micro‐coordination (i.e., communication about adolescent whereabouts). Moreover, more than half of adolescents (55.4%) communicated only infrequently and relatively briefly (less than an hour a day) with their parents online, but other adolescents (smaller subgroups) communicated much longer or more frequently.

### Almost all adolescents communicate with parents, but to a different degree

The smartphone provides the potential for continuous communication between parents and adolescents (Licoppe, 2004; Ribak, 2009). Before examining whether adolescents perceive this as a digital leash or a digital umbilical cord, it is essential to gain descriptive and ecologically valid insights into this online communication. The small body of research on parent‐adolescent online communication, mostly focusing on frequency, indicated that a little more than half of adolescents texted their parents at least once a day (e.g., Chang, 2015; Lenhart et al., 2010; Padilla‐Walker et al., 2012). In contrast, our study revealed that only 1.7% of adolescents communicated with their parents online every day and 14.4% every day or almost every day throughout the study period. Thus, the use of an extensive daily diary design shows that a smaller portion of adolescents communicate daily online with their parents than shown in previous cross‐sectional work.

In terms of duration, our findings unveiled that half of adolescents communicated with parents online for more than 10 min and the other half for less than 10 min. This result largely aligns with the findings of the only previous daily diary study that assessed the duration of online communication, which found that adolescents communicated online for approximately 15 min a day (Manago et al., 2020). Compared to the several hours of offline communication a day that adolescents have with their parents (Keijsers et al., 2010), our study suggests that for the vast majority of adolescents, online communication with parents is only a small extension or substitute for offline communication with parents.

However, this pattern did not apply to all adolescents. In fact, 11% of adolescents communicated for a more extended time with their parents online, ranging between 1 to 4 hours per day. The question remains why these adolescents (or parents) do so. One previous daily diary study indicated that adolescents with higher levels of mental health symptoms tended to seek more support than adolescents with lower levels of mental health symptoms (Jensen, George, et al., 2021). Also, adolescents who experienced more externalizing symptoms reported most parental control via online communication (Jensen, George, et al., 2021). While this may suggest that adolescents' mental health may be a factor to consider, caution should be warranted. Another study showed that depression can lead to overestimations of time spent on social media (Sewall et al., 2020). Overall, although smartphones provide the opportunity for adolescents and parents to connect anywhere and anytime, it does not happen all day and every day and it varies between adolescents.

### Parent–adolescent online communication mostly concerns micro‐coordination

One of the key questions of this study was to obtain a detailed understanding of the topics about which adolescents communicate online with their parents. By asking adolescents repeatedly, we were able to assess topics of parent‐adolescent online communication without recall bias and quantify the prevalence of topics, thereby extending previous qualitative research (e.g., Fletcher et al., 2018; Racz et al., 2015; Tulane et al., 2022). Indeed, adolescents and parents mostly used online communication for micro‐coordination, to inform their parents (on own initiative or upon request of parents) about, for instance, where they were and when they would be home. Furthermore, the two main categories of parent‐adolescent communication that were distinguished in a previous study, managerial and emotional communication (Fletcher et al., 2018), also emerged in the current study. Daily parent‐adolescent online communication concerned functional communication such as “Don't forget your appointment with the dentist tomorrow” and “Can you unload the dishwasher?” Adolescents also expressed feeling supported by parents who asked how they were doing or how their day was. The topics that adolescents and parents communicate about online thus appear to be an extension of their offline communication, aligning with the co‐construction theory (Subrahmanyam et al., 2006).

At the same time, our inductive approach provided several unexplored domains of parent‐adolescent online communication. For instance, adolescents reported to communicate online with their parents about fun things, such as music and sharing photos and videos. Intriguingly, parents did not only use online communication to check in on their adolescents (Jensen, George, et al., 2021), but some of the adolescents also did so with their parents. They monitored and supported their parents by inquiring about parents' whereabouts and well‐being; a relatively rare pattern which is to the best of our knowledge hitherto undescribed. The opportunity to seek out parental support and inquire about when to expect parents home (e.g., ‘Where are you and when are you home?’) is a unique novel opportunity for adolescents to be active agents in shaping the parent‐adolescent relationship and may indicate a more bidirectional micro‐coordination. Although these two newly emerged topics, adolescents monitoring and supporting parents, may indicate the realignment of the parent‐adolescent relationship, transitioning from a more authoritarian to a more egalitarian relationship (e.g., Branje et al., 2012; Grotevant & Cooper, 1986), for some adolescents it may also be less beneficial.

### Duration of communication decreases with age and boys communicate longer than girls

We found that age and gender explained some differences between adolescents in the profiles of parent–adolescent online communication, but not living situation and autonomy. Younger adolescents communicated longer with parents online than older adolescents. This seems to be in line with a general decrease in the level of offline communication with parents during adolescence (Keijsers & Poulin, 2013; Lionetti et al., 2019). Additionally, our findings unveiled that adolescents who communicated online with their parents for a longer period were more likely to be boys compared to adolescents who communicated shortly with their parents online (both infrequent and frequent). This contradicts earlier work that showed that age and gender did not relate to the duration of parent‐adolescent online communication in daily life (Manago et al., 2020). Moreover, it indicates that results on the frequency of parent‐adolescent online communication do not necessarily translate to the duration of parent‐adolescent online communication. Furthermore, previous research suggested that adolescents with more autonomy, and those who live apart from both parents would report more frequent and longer online communication with parents (Manago et al., 2020; Padilla‐Walker et al., 2012; Racz et al., 2015), but our findings did not support this finding. An important difference here is that previous studies mostly focused on the frequency of parent‐adolescent online communication or solely on the duration, while our study used a combination of frequency and duration to obtain the profiles of parent–adolescent online communication.

### Limitations and avenues for future research

This study has described in much detail which patterns exist in parent–adolescent online communication. However, it did not distinguish between one‐on‐one chats or group chats with the family, nor did it identify whether the communication was initiated by the parent or adolescent. Future work is needed to differentiate between types of chat because topics may differ between one‐on‐one and group chats. Also, the lack of differentiation may result in adolescents under‐ or overreporting communication with parents. In addition, future research needs to investigate the underlying dynamic processes, such as who initiates, and who ends the communication (e.g., Hayes et al., 2004). This could be investigated, for instance, by distinguishing parental solicitation from (unsolicited) adolescent disclosure and by examining how responses by either the adolescent or parents may enhance or inhibit future communication patterns.

Such granular insights in parent–adolescent online communication require an even more micro level assessment (hour‐to‐hour) than our daily diary study. Promising avenues for future research are Experience Sampling Methods or the analysis of WhatsApp chats or text messages exchanged. The latter has been done in earlier work between parents‐adolescents and parents‐emerging adults (Ehrenreich et al., 2020; Jensen et al., 2023; Jensen, Hussong, et al., 2021). Such methods allow for more insights into the flow of online communication as well as into more detailed content and how adolescents and parents react to each other. Examination of text messages or chats would further enable the coding of parent solicitation and adolescent disclosure as well as using text analysis to gain a more detailed insight into topics of parent‐adolescent online communication (see for a modeling example Verbeij et al., 2024). Additionally, analyzing text messages or chats (in combination with self‐report) would help to gain more insight into the accuracy of the measurement. Asking adolescents to indicate how long they communicated online with parents over a day may be hard to estimate and be prone to error.

Similarly, our study was unable to assess the developmental consequences of online communication with parents. Although our findings indicated that most adolescents communicate shortly with their parents, some do it longer. The question remains whether prolonged parent‐adolescent online communication is positive or negative for adolescents. The extent to which it for instance impacts parent–adolescent closeness and relationship quality remains unknown and requires more investigation. The ability of parents *and* adolescents to contact each other throughout the day may also benefit their relationship, providing new and more extended opportunities to stay connected (Jensen, George, et al., 2021; Vaterlaus et al., 2019). However, the finding that some adolescents also monitor and support their parents raises new important theoretical questions. Does it reflect a healthy and normative transformation of the parent–adolescent relationship? Or is it potentially a sign that adolescents take high levels of responsibility for their parents' well‐being (Hooper, 2007)? Although replication of our results is needed, future studies may want to include both parent‐adolescent online and offline communication in relation to relationship quality, closeness, well‐being, or psychopathology to gain a more thorough understanding of parent‐adolescent communication and the role of online communication as well as differences between individuals.

Future research may need to address at least two several other important issues. First, an unanswered question is how adolescents perceive the continuous potential of communication with parents (i.e., connected presence, Licoppe, 2004). While smartphones provide the opportunity to communicate online with each other continuously, it remains unknown what matters more for parents and adolescents: the possibility to communicate with each other when needed or wanted or the actual online communication? Future work may want to assess the impact of the possibility of online communication.

Second, insights into how parents and adolescents perceive online communication are lacking. Do adolescents feel supported or controlled? Previous work on parent‐adolescent offline communication has indicated that how adolescents perceive social interaction (i.e., quality) is more strongly related to adolescent well‐being than how often it takes place (i.e., quantity; Liu et al., 2019). Some previous work on online communication, analyzing coded content of texts between parents and emerging adult, showed, for instance, that both quality and quantity of online communication were unrelated to perceived parents' autonomy support (Brown et al., 2024). The quantity of online communication was related to emerging adults' perceptions of their mothers digital support (Jensen, Hussong, et al., 2021). However, these studies did not address adolescents' daily perceptions of online communication with parents. Future work may want to include assessments of how adolescents experience parent‐adolescent online communication in addition to quantitative aspects of parent‐adolescent online communication such as frequency and duration. It may allow for testing hypotheses such as whether the extent to which adolescents perceive chatting with parents as positive or negative, supportive or controlling may matter for their well‐being, (e.g., Bülow et al., 2022). This avenue for future research also aligns with a more general call from social media scholars to pay more attention to experiences on social media instead of solely focusing on the frequency of use (Valkenburg et al., 2022).

## CONCLUSION

Smartphones have become an integral part of family life and enable parents and adolescents to communicate with each other anywhere and anytime. By assessing the frequency, duration, and topics of parent–adolescent online communication over a span of 100 days, this study demonstrated that almost all adolescents communicate online with their parents. On average, adolescents communicated with their parents on 43% of the days, for an average of 20 min a day. However, differences were found, resulting in five distinct profiles. Although almost 90% of adolescents communicated online with their parents for a short period, other adolescents communicated longer with their parents. Younger adolescents and boys communicated longer than older adolescents and girls. Most online communication between parents and adolescents concerned monitoring and micro‐coordination. However, some adolescents also monitored and supported their parents through online communication. This fine‐grained descriptive study calls for future investigations to gain insight into the underlying communication dynamics as well as the impact of the possibility of staying in contact online for parent–adolescent relationship quality and both adolescent and parent well‐being.

## FUNDING INFORMATION

This preregistered study was funded by an NWO Spinoza Prize awarded to Patti M. Valkenburg by the Dutch Research Council (NWO). Additional funding was received from a VIDI grant (NWO VIDI Grant 452.17.011) awarded to Loes Keijsers by the Dutch Research Council (NWO). The research was further supported by a grant from the European Research Council (ERC; 101043536) awarded to Loes Keijsers.

## CONFLICT OF INTEREST STATEMENT

The authors declare no conflicts of interest.

## Supporting information


Data S1.

## Data Availability

The R and M*plus* syntaxes necessary to reproduce the analyses presented here are publicly accessible at OSF: https://osf.io/2r5xd. The code was checked and run by an independent co‐pilot. The procedure and materials necessary to replicate the findings are also available at OSF: https://osf.io/k47ta. More information on the measures and pre‐registered analyses can be found in the preregistration at OSF: https://osf.io/9vyfr. The data underlying this article can be found on Figshare: https://doi.org/10.21942/uva.27854697.
