# Supplementary material for: Parent–adolescent communication in a digital world: A 100‐day diary study
Source: Child Dev. 2024 Nov 22;96(2):736–51. doi: 10.1111/cdev.14203 (PMC11868674; doi:10.1111/cdev.14203)
Supplement: Supplementary file 1 — Data S1. [file CDEV-96-736-s001.docx]

**Parent-adolescent communication in a digital world:**

**A 100-day diary study**

**Supplementary Materials**

**Appendix 1.**

Detailed information about the youth advisory discussion sessions and pilot study

*Youth advisory discussion sessions*

Youth advisory discussion sessions were organized with nine adolescents (13-17 years old; 66.7% girls) to discuss with adolescents how to make the study as enjoyable as possible and how to set up the study procedure. We also discussed the content of each item in the daily questionnaire and checked comprehension and interpretation. Relevant for the current study is the estimation of the duration of parent-adolescent online communication. Adolescents themselves did not mention that they found it difficult to report this.

*Pilot study*

A pilot study was performed to test multiple things: the technological performance and stability of the m-Path app, within-person variability, feasibility of sampling scheme, and comprehension of items. 26 adolescents (13 boys and 13 girls; 13-17 years) participated in a pilot daily diary study of 14 days. Adolescents received a notification at 8.30 PM to complete the daily questionnaire and at 9.15 PM a reminder was sent if the questionnaire was not yet completed. If adolescents indicated that they communicated online with their parents, they received several follow-up questions and one follow-up question concerned the topic of communication. This was an open question and adolescents could fill in own topic(s). The given answers on this question were used to create the a-priori response categories.

Overall, the pilot study showed technical stability of the m-Path app and that the instruments showed sufficient within-person variability. The sampling scheme fitted the agendas but older adolescents indicated that they would appreciate one reminder later in the evening. We added an extra reminder at 10.00 PM in the full study.

Adolescents were asked afterwards in short interview if they had trouble with answering items or answering options. They did not indicate having difficulties with answering how long they communicated online with their parents.

**Appendix 2.**

Measurement of demographic variables

***Gender***

We assessed adolescents’ gender using one item: “I am …” with three response categories: (1) a boy, (2) a girl, (3) fill in. Adolescents could insert text when they selected “fill in”. Four adolescents responded to “fill in”.

***Age***

We assessed adolescents’ age by asking their date of birth in the baseline questionnaire. Age was calculated by subtracting date of completing the baseline from date of birth.

***Family living situation***

We assessed adolescents’ family living situation using one item: “With whom do you live most days of the week?” with eight a-priori response categories: (1) with both of my parents, (2) partly with one parent, partly with the other, (3) with my mother and her partner, (4) with my mother, (5) with my father and partner, (6) with my father, (7) with others (e.g., foster parents, other family), (8) by myself. For the analyses, the categories were recoded to living with both parents (1) and other living situations (2; 25.7%) taking all the other 7 categories together.

**Appendix 3.**

Fit measures latent profile sensitivity analyses with five profile including adolescents without parent-adolescent online communication (*N* = 479 adolescents)

| # of classes | BIC | AIC | LL (parameters) | LMR-LRT (*p*) | Entropy | Number (percentage) per profile) | | | | | |
| --- | --- | --- | --- | --- | --- | --- | --- | --- | --- | --- | --- |
|  |  |  |  |  |  | 1 | 2 | 3 | 4 | 5 | 6 |
| 1 | 4887.402 | 4870.716 | -2431.358 | - | - | 479 (100%) |  |  |  |  |  |
| 2 | 4547.246 | 4518.044 | -2252.022 | 340.293 (.433) | 0.991 | 461 (96.2%) | 18 (3.8%) |  |  |  |  |
| 3 | 4418.490 | 4376.773 | -2178.386 | 139.725 (.261) | 0.972 | 425 (88.7%) | 41 (8.6%) | 13 (2.7%) |  |  |  |
| 4 | 4280.294 | 4226.062 | -2100.031 | 148.680 (.010) | 0.985 | 421 (87.9%) | 44 (9.2%) | 11 (2.3%) | 3 (0.6%) |  |  |
| 5 | **4181.806** | **4115.058** | **-2041.529** | **111.008 (.003)** | **0.922** | **271 (56.6%)** | **155 (32.4%)** | **40 (8.4%)** | **10 (2.1%)** | **3 (0.6%)** |  |
| 6 | 4150.666 | 4071.404 | -2016.702 | 47.110 (.090) | 0.922 | 267 (55.7%) | 149 (31.1%) | 42 (8.8%) | 10 (2.1%) | 8 (1.7%) | 3 (0.6%) |

*Note.* Class counts and proportions are based on their most likely class membership. Stable class solution was tested by 800 and 160 random starts. To test the stability of the chosen solution, models were run again with doubled starting values (1600 320) and model results were replicated. Bolded values represent the final profile solution (based on LMR-LRT).

The table shows that the BIC of the five-profiles solution was lower than the BIC of the four-profiles solution and the LMR-LRT confirmed that the five-profiles solution was significantly better than the four-profiles solution. Even though the six-profiles solution had an even lower BIC, the LMR-LRT was not significant, which indicates that this solution did not fit the data better than the five-profiles solution.

**Appendix 4.**

Results of preregistered approach whether four individual characteristics predicted profile membership regarding parent-adolescent online communication by saving out profile membership

We saved class membership to compare the profiles of parent-adolescent online communication. For the comparison of groups, we preregistered that we would interpret differences between profiles with more than 40 adolescents (based on power analysis). As one profile had 39 adolescents, which is a minor deviation from that cut-off, we decided to take this profile into account. Therefore, we interpret differences between adolescents in the three largest groups: *infrequent short communication*, adolescents with *frequent short communication*, and adolescents with *medium-long communication*. The chi-square difference test including gender only included boys and girls, as the non-binary category was too small to be included (4 persons).

Gender predicted profile membership (χ^2^ (2, *N* = 447) = 6.57, *p* = .037; Figure 3). Post-hoc analyses with Bonferroni adjustments indicated that adolescents with *medium-long communication* were more likely to be boys than adolescents with *frequent short communication* (*p* = .011). Family living situation (χ^2^ (2, *N* = 451) = 2.44, *p* = .296) did not predict parent-adolescent online communication profile membership. Since the normality assumption was violated, non-parametric tests were used for age and autonomy. Age predicted profile membership (Kruskal-Wallis test, *H*(2) = 6.03, *p* = .049). Adolescents with *medium-long communication* were younger than adolescents with *infrequent short communication* (Figure 4; post-hoc pairwise Wilcoxon tests with multiple testing correction Benjamini-Hochberg, *p* = .0497). Autonomy did not predict profile membership (Kruskal-Wallis test, *H*(2) = 2.68, *p* = .261).

*Gender predicting profile membership, with more adolescent boys in profile 3 (medium-long communication) than in profile 2 (frequent short communication)*

*
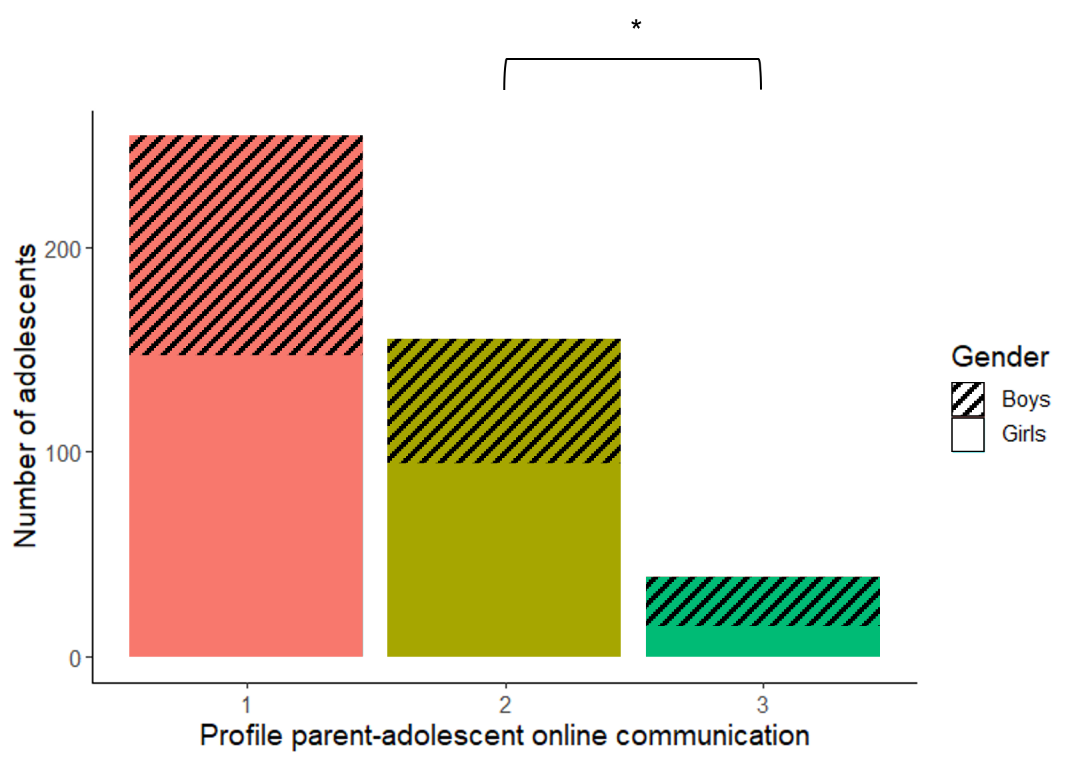
*

*Age predicting profile membership, adolescents in profile 3 (medium-long communication) were younger than adolescents in profile 1 (infrequent short communication)*
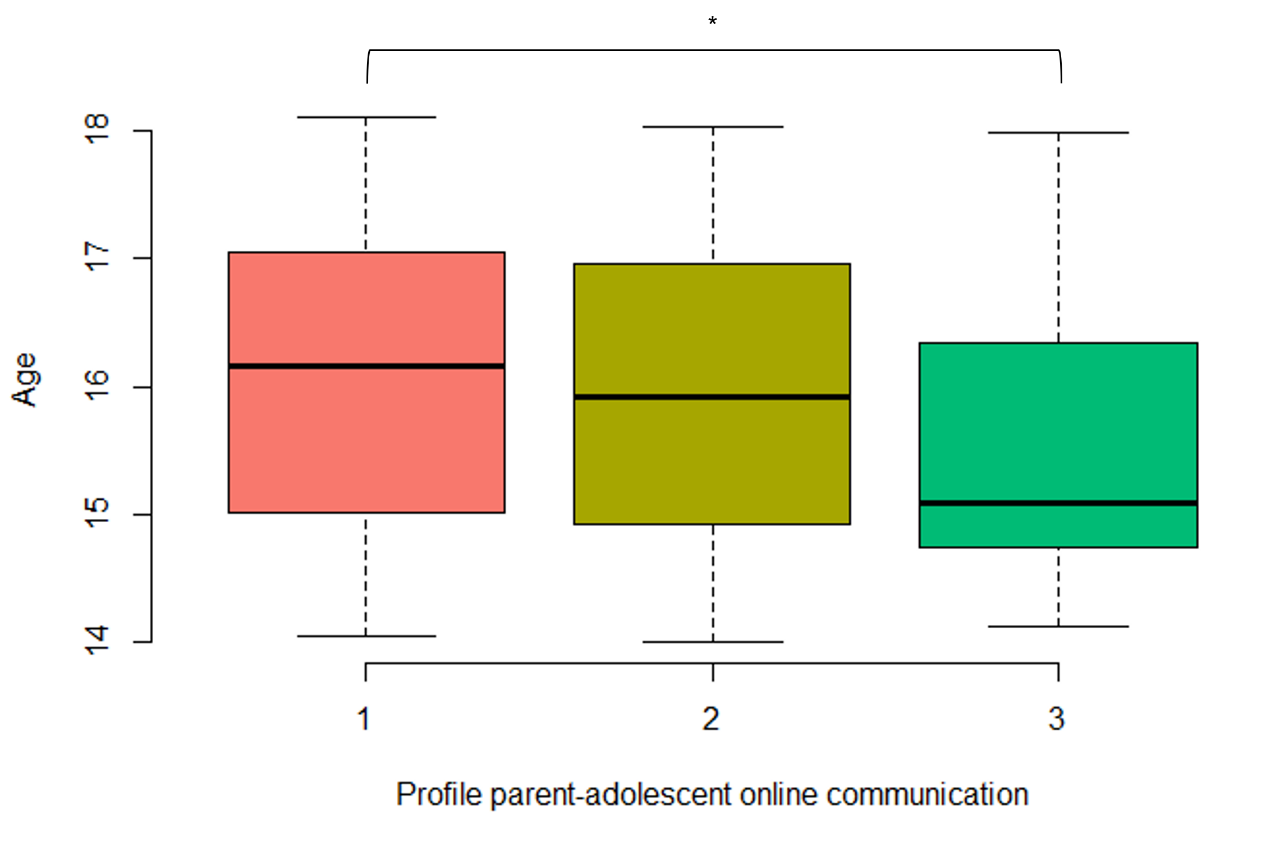


**Appendix 5.**

Scree plot and figures of all profile solutions, except the final five-profile solution

*Scree plot of BIC-values of all tested profile solutions*


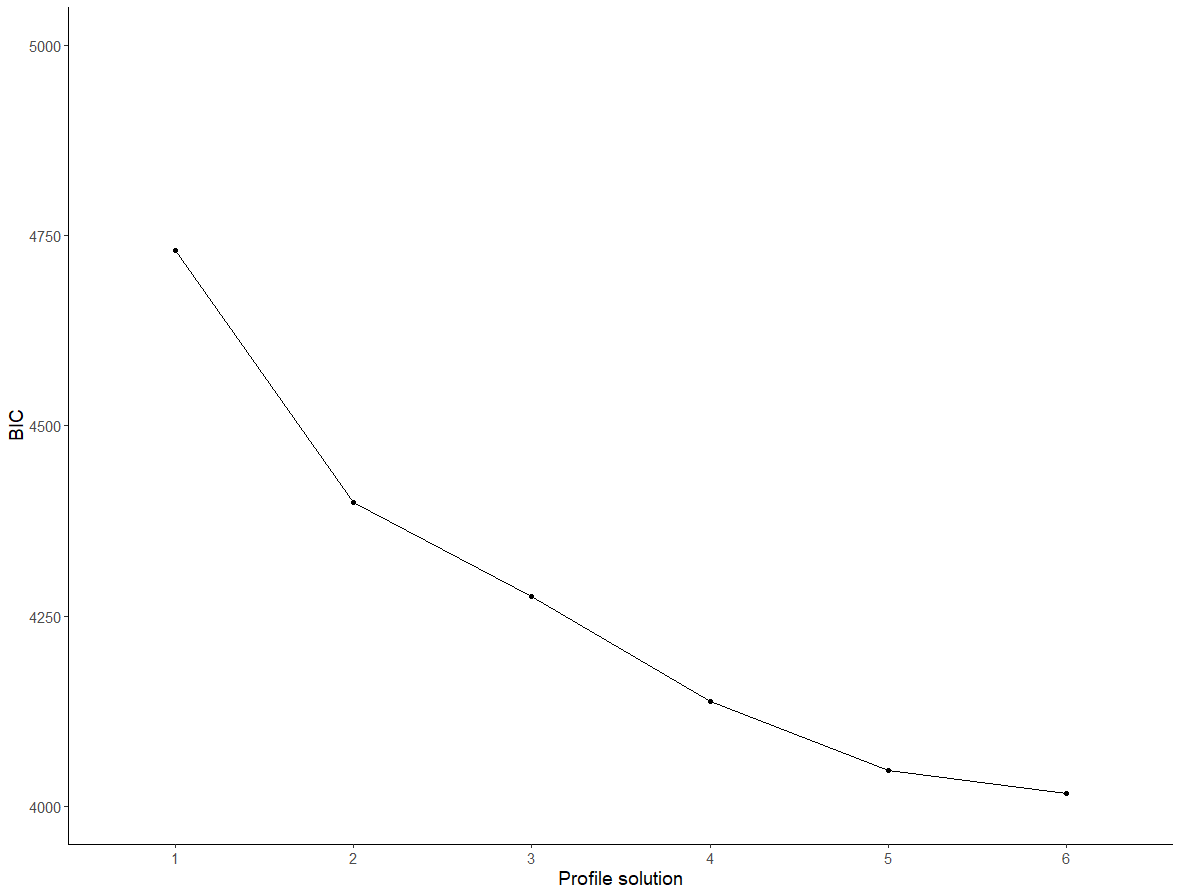


*Two profile solution*

*
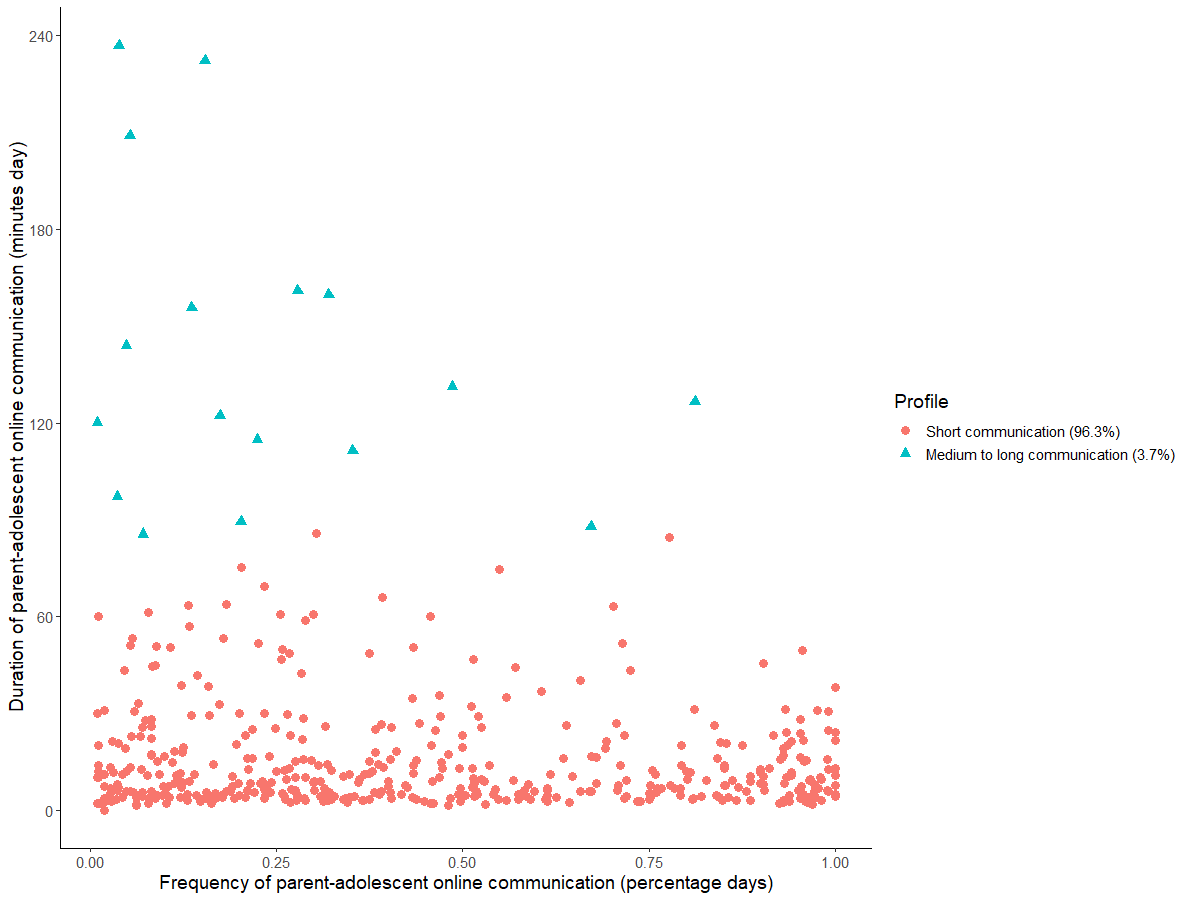
*

*Three profile solution*

*
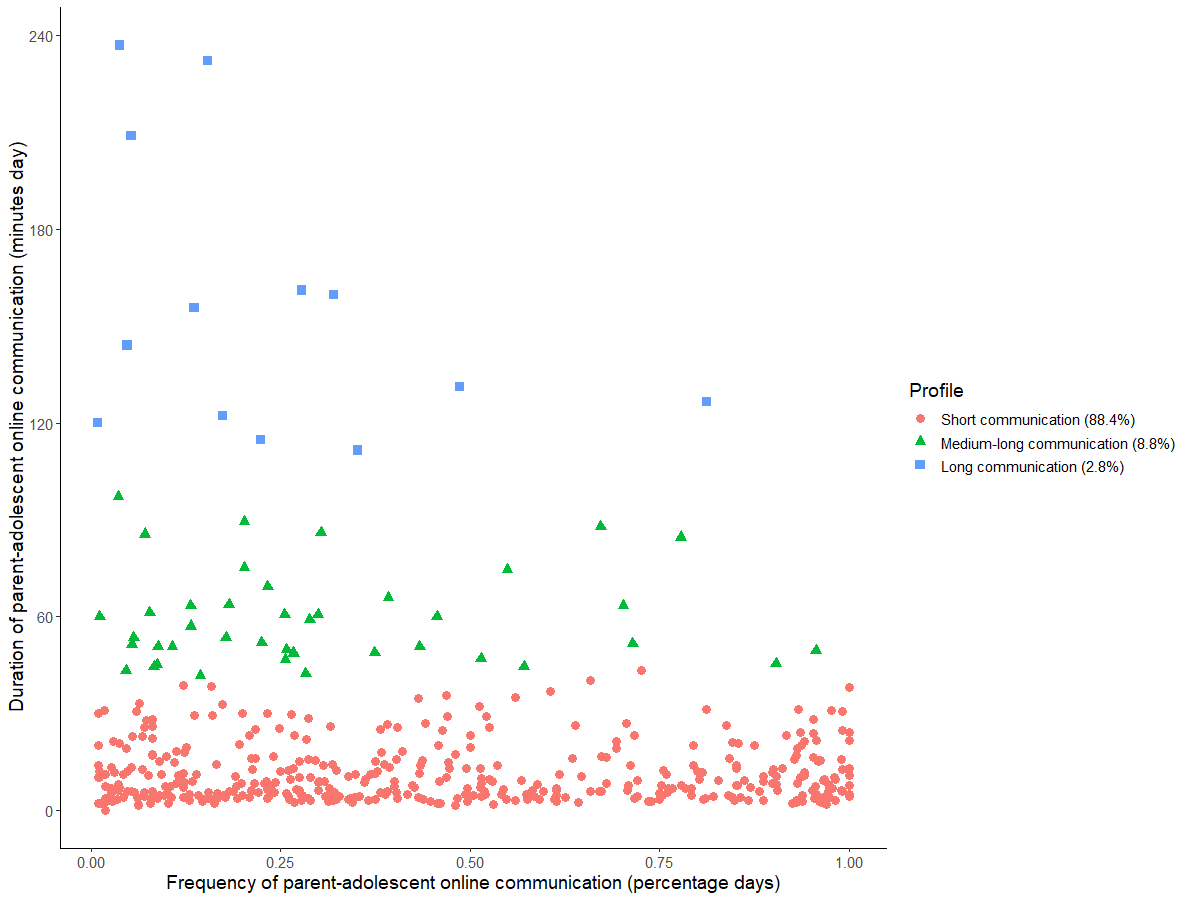
*

*Four profile solution*


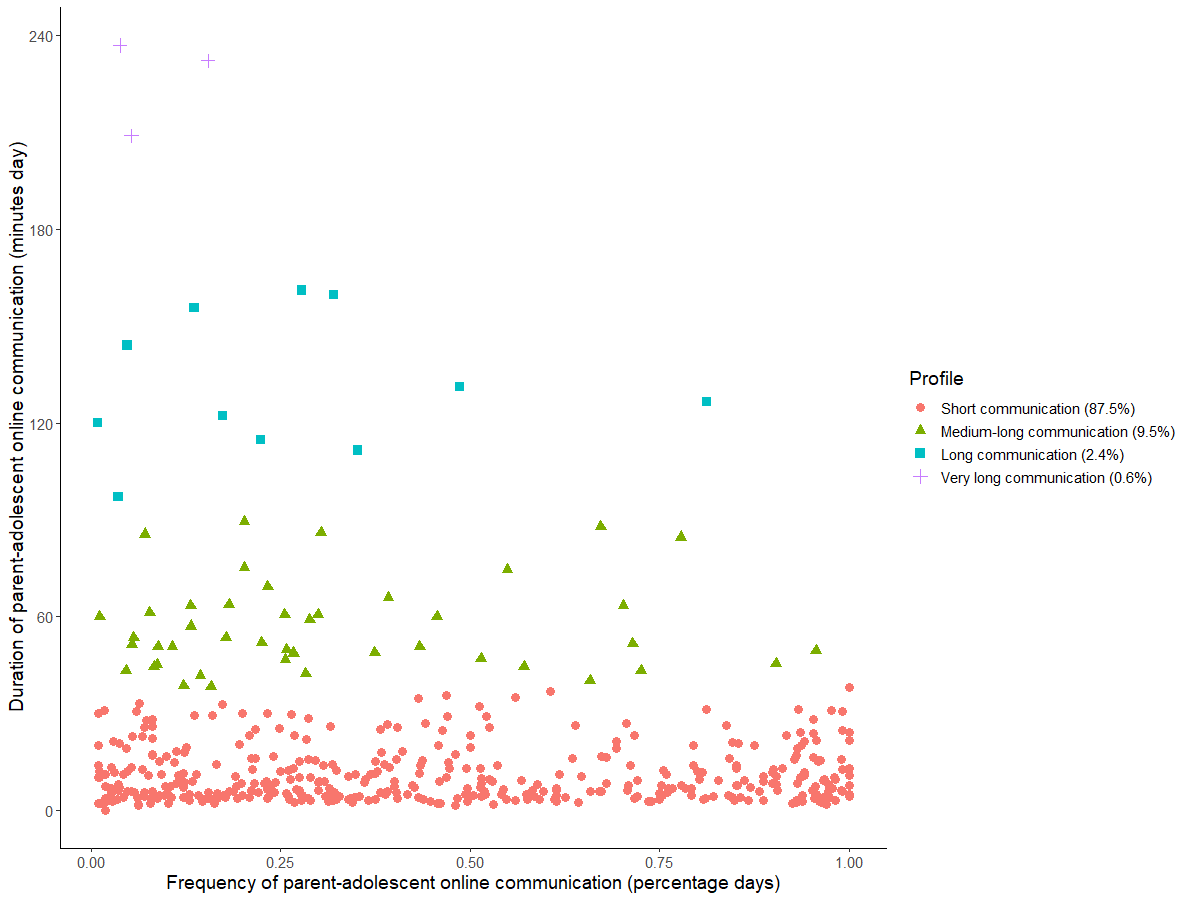


*Six profile solution*

*
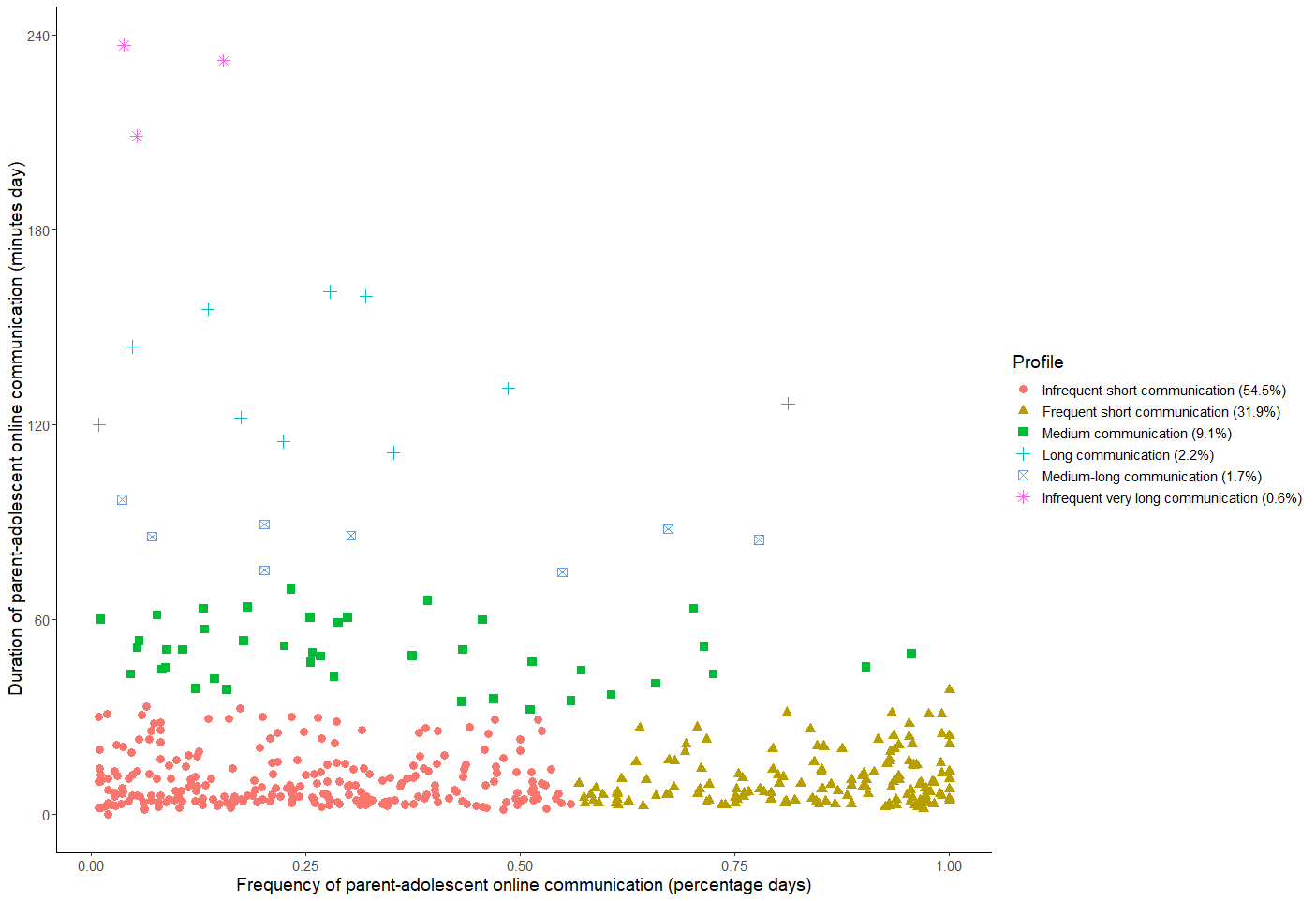
*

**Appendix 6.**

Details and results of sensitivity analyses

*Sensitivity analysis to check the potential influence of flagged observation.*

During data cleaning, some observations were flagged due to wrong or inconsistent time or date, because the variance of the first four variables was zero, due to unlikely or wrong values (e.g., chatting more than 20 hours a day), or careless responding in open answers. This concerned 1,004 observations, of which 754 observations included parent-adolescent online communication. We excluded these flagged observations and tested whether these values influenced our final model solution and recalculated the person-mean frequency and duration of parent-adolescent online communication. Rerunning the five-profile solution with random starts 800 and 160 provided similar profiles with a similar interpretation; replicating the main findings in these sensitivity analyses. However, the LMR-LRT was not significant, *p* = .454 (see below for fit indices and figure of profile solution). Therefore, we also reran the four-profile solution. The BIC was lower and LMR-LRT was significant, indicating that the four-profile solution was a better model fit than the three-profile solution (see below for fit indices and figure of profile solution). The figure shows that two small profiles seem to have merged: the infrequent medium-long communication and infrequent long communication.

We ran four models, one for each predictor, to assess whether these predicted profile membership using the 3-step procedure in M*plus*. Comparisons between all four profiles for all four predictors are presented in the table below. The two smallest profiles were not considered for interpreting differences due to their small size. Age, autonomy, and living situation did not predict profile membership. Thus, although the profile solution differed slightly from our original findings, the main conclusions remained the same.

Fit measures latent profile sensitivity analyses with five profile without flagged observations (*N* = 463 adolescents)

| # of classes | BIC | AIC | LL (parameters) | LMR-LRT (*p*) | Entropy | Number (percentage) per profile) | | | | | |
| --- | --- | --- | --- | --- | --- | --- | --- | --- | --- | --- | --- |
|  |  |  |  |  |  | 1 | 2 | 3 | 4 | 5 | 6 |
| **4** | **4220.242** | **4166.451** | **-2070.226** | **119.881 (.018)** | **0.913** | **282 (60.9%)** | **155 (33.5%)** | **22 (4.6%)** | **4 (0.8%)** |  |  |
| 5 | 4128.339 | 4062.135 | -2015.068 | 104.634 (.454) | 0.920 | 260 (56.2%) | 152 (32.8%) | 36 (7.8%) | 11 (2.4%) | 4 (0.9%) |  |

*Note.* Of one adolescent, all observations including parent-adolescent online communication were flagged. Class counts and proportions are based on their most likely class membership. Random starts 800 and 160.

|  | Age  *Est* (*p*-value) | Gender^a^  *Est* (*p*-value) | Autonomy  *Est* (*p*-value) | Living situation  *Est* (*p*-value) |
| --- | --- | --- | --- | --- |
| Infrequent short vs frequent short | -0.071 (.460) | 0.292 (.199) | 0.130 (.505) | 0.354 (.170) |
| Infrequent short vs medium-long | -0.148 (.462) | -0.391 (.199) | 0.458 (.166) | 0.423 (.284) |
| Infrequent short vs infrequent very long | -0.664 (.347) | **-18.792 (<.001)** | 1.475 (.123) | 0.538 (.663) |
| Frequent short vs medium-long | -0.077 (.706) | -0.683 (.148) | 0.328 (.338) | 0.069 (.865) |
| Frequent short vs infrequent very long | -0.593 (.402) | **-19.085 (<.001)** | 1.345 (.162) | 0.185 (.881) |
| Medium-long vs infrequent very long | -0.516 (.479) | **-18.402 (<.001)** | 1.017 (.306) | 0.116 (.928) |

*Note.* The first category that is mentioned is the reference category. Bolded values indicate significant differences.  ^a^Model only included boys and girls to resemble original analysis.

Figures of five and four profile solution of sensitivity analyses without flagged observations

*Five profile solution*

*
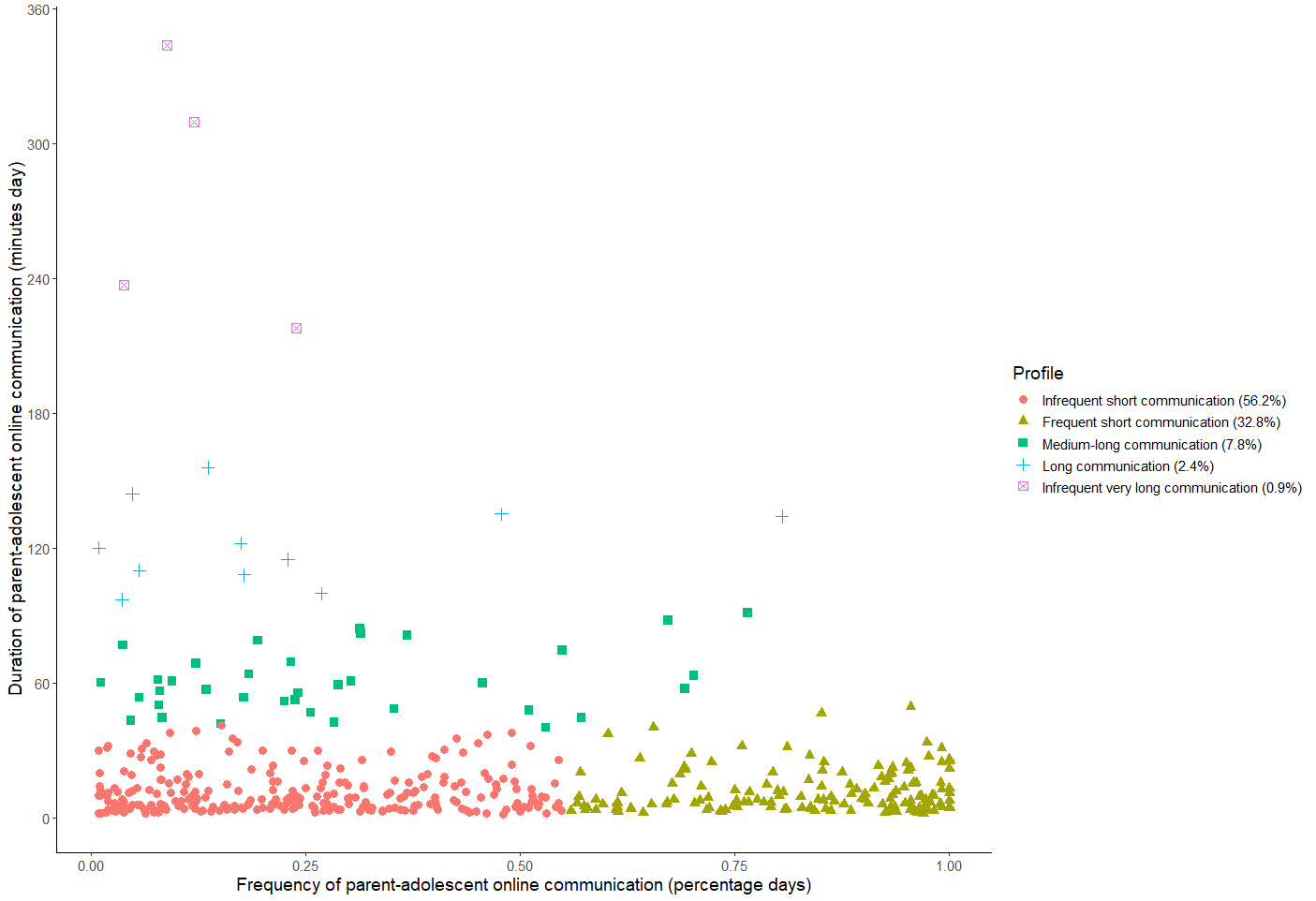
*

*Four profile solution*


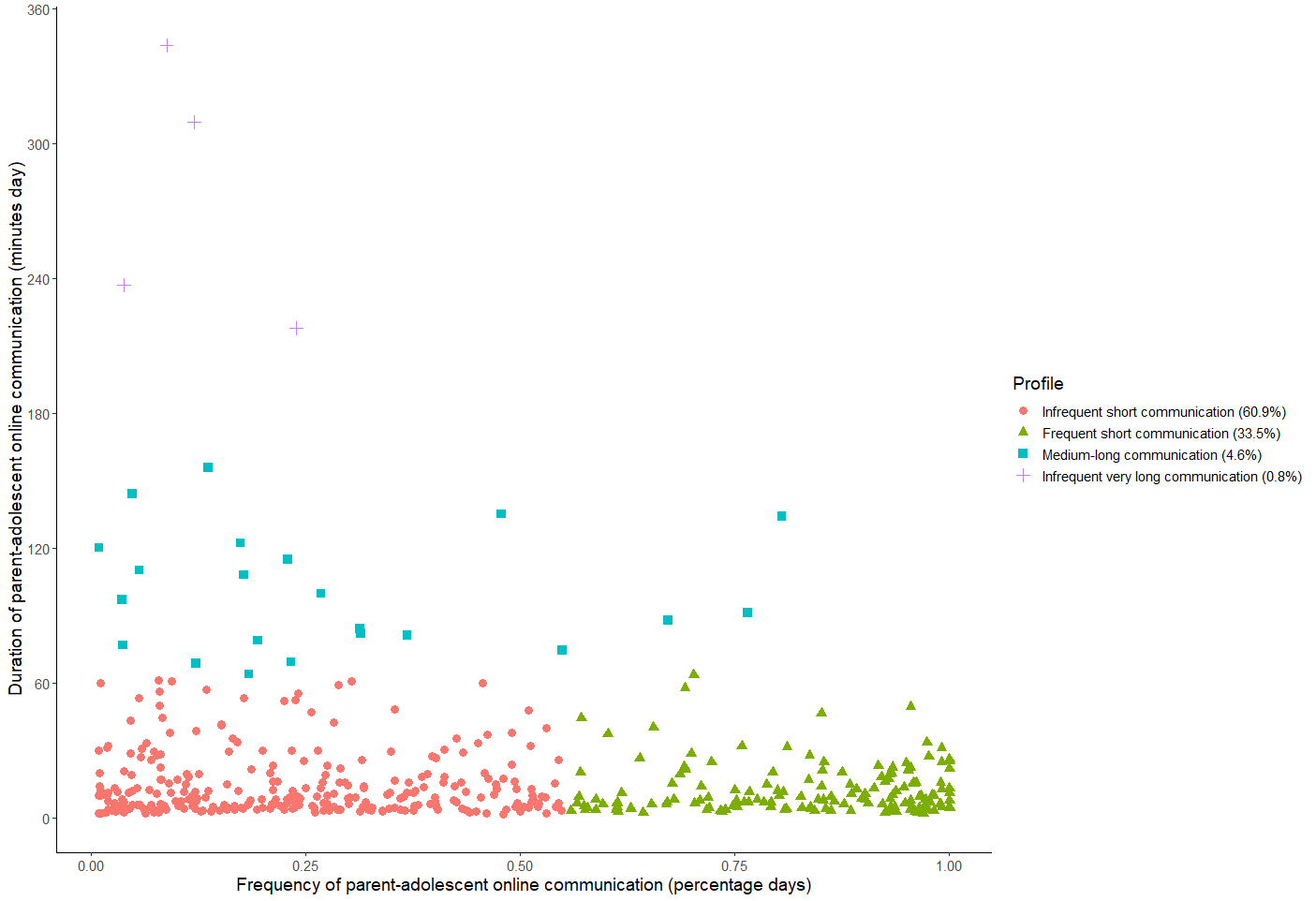


*Sensitivity analyses to treat autonomy as distal outcome and accounting for demographic differences*

To compare autonomy scores across profiles and account for gender, age, and family living situation that may impact adolescents’ perceptions of autonomy, we used the BCH approach (Vermunt, 2017) and treated autonomy as distal outcome. Results indicated that autonomy scores did not significantly differ across the profiles. Medium-long communication vs frequent short communication was *p* = .428, frequent short communication vs infrequent short communication was *p =* .522, and medium-long communication vs infrequent short communication was *p* = .218.

**References**

Vermunt, J. K. (2017). Latent class modeling with covariates: Two improved three-step approaches. *Political Analysis*, *18*, 450-469. <https://doi.org/10.1093/pan/mpq025>
